# Cerebral Venous Sinus Thrombosis during Everest Expedition: A Case Report and Review of the Literature

**DOI:** 10.1155/2016/8314040

**Published:** 2016-10-31

**Authors:** P. Khanal, L. Thapa, A. M. Shrestha, S. Bhattarai, D. Sapkota, N. Sharma, U. P. Devkota

**Affiliations:** ^1^Dickinson College, Carlisle, PA, USA; ^2^Department of Neurology, National Institute of Neurological and Allied Sciences, Kathmandu, Nepal; ^3^Department of Neurosurgery, National Institute of Neurological and Allied Sciences, Kathmandu, Nepal

## Abstract

Cerebral venous sinus thrombosis (CVST) is a rare but serious disorder that is associated with a poor clinical outcome. We report a 35-year-old man who had a severe headache and diplopia while climbing Mount Everest. His MR venography showed right transverse and right sigmoid sinus thrombosis. He improved on anticoagulant and symptomatic measures. Cerebral venous sinus thrombosis at high altitude is discussed.

## 1. Introduction

Cerebral venous sinus thrombosis (CVST) refers to complete or partial occlusion of either the main sinus/sinuses or the feeding cortical veins leading to secondary effects of vascular congestion and focal or generalized neurological deficits [1]. It is a rare but serious disorder, and at a high altitude this disorder is reported meagerly [2, 3]. Very few cases are diagnosed for various reasons and due to the lack of numerous analogous cases; this disorder can often be misdiagnosed [4, 5]. In Nepal, increased awareness of this condition amongst physicians and availability of investigation facility in recent years have led to early diagnosis and favorable outcome in patients with CVST.

## 2. Case Report

**Figure 1:**
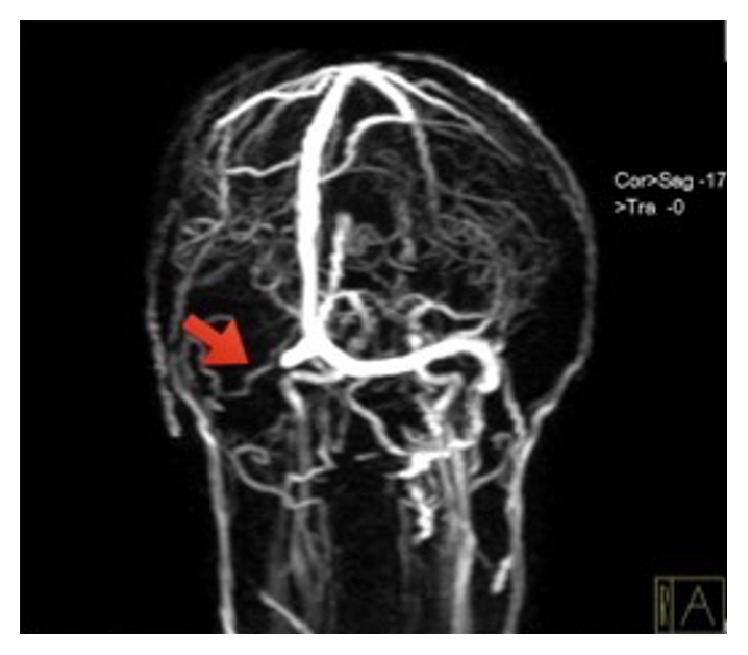
MRI venography showing right transverse and sigmoid sinus thrombosis (arrow).

A 35-year-old man, trekker, and guide by profession had presented with a sudden onset of severe right hemicranial headache since six days. His headache started immediately after returning from Mount Everest (8848 meters) expedition for the fourth time. It was associated with vomiting, increased thirst, and diplopia. He had no significant past medical or surgical illnesses. He is a heavy drinker and a smoker. Examination revealed normal Glasgow coma scale, bilaterally reactive pupils, papilledema with retinal hemorrhages, and a right-sided VIth cranial nerve (CN) palsy. His motor and sensory examination were normal. There were no signs of meningeal irritation. Investigations showed hemoglobin: 18.3 gm%, hematocrit: 56.5, and normal erythrocyte sedimentation rate. His white blood cell count, platelets, bleeding time, clotting time (prothrombin time), and activated partial thromboplastin time were normal. His blood glucose, serum electrolytes, renal function, liver function, and routine urine examination were normal. Tests for HIV I/II, HBsAg, and HCV were negative. His FDP (fibrin/fibrinogen degradation products) was normal (<200 ng/ml) and d-dimer was elevated (963 ng/ml; normal <500 ng/ml). His cerebrospinal fluid study was also normal except for increased opening pressure. His plasma protein C activity was normal (103%; normal: 67–195%); however, he had low plasma protein S activity (49%; normal: 55–123%). MR venography revealed nonvisualization of right transverse and sigmoid sinuses s/o right transverse and sigmoid sinus thrombosis (Figure 1). He was treated with low molecular weight heparin and intravenous mannitol (2 g/kg) over 20 mins for 3 days for raised intracranial pressure and symptomatic measures for his headache and vomiting. His headache subsided and diplopia was improving; however, his diplopia was persistent at discharge.

## 3. Discussion 

In adults, the incidence of CVST has been estimated to be as high as 3-4 cases per million [6] and it accounts for 0.5% of all stroke cases [7]. Indeed, predisposing factors can be identified in up to 80% of cases [8]. Common etiologies include head injury [9], infections [10], oral contraceptive pills [11], inflammatory disorders, hypercoagulable disorders [8], dehydration [12], and malignancies [10].

Very little is known about stroke at high altitude and most of the reports are limited to isolated cases of the ischemic neurological syndrome. Although high altitude is one of the unusual causes of CVST amongst many [13], the occurrence of CVST due to the ascent to high altitude is a well-documented phenomenon recently [2]. CVST has been seen in people who climb to high altitudes of about 5000 meters (range 2200–5500 meters). Volume depletion and polycythemia are implicated as a plausible explanation for CVST at high altitude [14]. Recently numerous factors like hypoxia, heat stress and hyperthermia [15], extremely low temperature [16], immobilization during long distance travel [17], and stress related to occupational hazards at high altitude (avalanche risk and unpredictable weather) are suggested to influence the increased risk for thrombosis at high altitude. This scenario coincides with our case who was diagnosed with CVST after he returned from climbing Mount Everest (8848 meters) for the fourth time. To note, many mountain climbers had lost their lives because of avalanche during recent earthquake (April 25, 2015), the worst disaster in Nepal, which has been a huge factor for high stress amongst mountain climbers in Nepal. Our patient had this stress as he had climbed Everest after the disaster. A small number of similar cases have been reported earlier attributing CVST to high altitude, including one from our center as well [18]. Combinations of risk factors may play a role in the causation of CVST at high altitude. Our case had dehydration, polycythemia, decreased protein S activity, and history of an ascent to high altitude. Ascent to high altitude in presence of hereditary thrombophilia (like protein S deficiency in our case) may lead to a widespread thrombosis (CVST and deep venous thrombosis of lower limb) in a patient like one described by Nair and his colleagues [19]. Our case did not have deep venous thrombosis of lower limbs, upper limbs, or pulmonary embolism. Fujimaki et al. had described a similar case in 1986 and, interestingly, the patient was in Nepal and had climbed 3161 meters on the way to Everest [3]. Various hereditary thrombophilic conditions like protein C deficiency and Factor V Leiden mutation leading to CVST in a high altitude climber have been reported [20]. The history and clinical and investigation findings suggest a multifactorial etiology of CVST in our case.

CVST is a challenging condition due to its wide range of clinical presentations [18, 21]. Various symptoms including a severe headache [22], abnormal vision, fainting or loss of consciousness, weakness of face and limbs on one side of the body, and seizures may occur. Our patient had a severe headache and diplopia due to the right VIth CN palsy. These symptoms are caused by raised intracranial pressure. The mechanism of VIth CN palsy with increased intracranial pressure has been attributed to the nerve stretching in its long intracranial course or compression against the petrous ligament or the ridge of the petrous temporal bone. Some authors, however, believe that it is more likely to be the mechanical effects of backward brain stem displacement [23]. Interestingly, vascular lesions affecting the VIth CN triggered by hypoxia, dehydration, coagulation defects, polycythemia, or vascular spasms at high altitude have been proposed [14].

As there is increasing awareness of this condition at our institute, we screen many patients having a headache for possible CVST, irrespective of their address (geographical location), medication use, or occupation because we have realized in all cases etiology may not be evident.

It was easier for us to suspect CVST in our case because of the classical clinical description and examination findings. We treated our patient with IV fluids, mannitol, low molecular weight heparin, which was later changed to warfarin, and symptomatic treatment for a headache. Both the duration and use of anticoagulation in CVST are an area of controversy because of the lack of strong evidence; however, current standard practice is to use anticoagulation if no contraindication exists [24]. Although there are no randomized controlled trials to support use of mannitol in CVST, it definitely gives time before more specific measures can be instituted. However, it is important to understand that osmotic substances may be harmful in venous obstruction, as they are not as quickly eliminated from the cerebral circulation as in other conditions [25]. So far, our experience with mannitol in CVST has been satisfactory. His headache subsided; however, his diplopia was persistent at discharge. The recovery of VIth CN paresis is obviously not expected so early. Studies have shown the spontaneous recovery time of approximately 3 months in half of the patients with VIth CN palsy [26, 27].

With the early diagnosis and treatment, 80% of the patients with CVST have a favorable prognosis with full recovery at around 6 months [28]. Coma and intracerebral hemorrhage when present are known to significantly affect the outcome [29]. Our patient did not have such poor prognostic markers, and as he had shown remarkable improvement in his symptoms, we believe that he will do well on follow-up.

## 4. Conclusion

Mount Everest is still the ultimate mountaineering adventure and many people around the world are attracted to it. CVST is increasingly diagnosed in Nepal in patients climbing high altitude. The cause of CVST seems to be multifactorial. Hydration is essential to prevent CVST. Screening for prothrombotic states before visiting high altitude, if feasible, may prevent this serious but treatable disorder at high altitude.
